# Fibronectin and Its Applications in Dentistry and Periodontics: A Cell Behaviour Conditioner

**DOI:** 10.7759/cureus.30702

**Published:** 2022-10-26

**Authors:** Unnati Shirbhate, Pavan Bajaj, Jinnie Pandher, Khushboo Durge

**Affiliations:** 1 Periodontics and Implantology, Sharad Pawar Dental College, Datta Meghe Institute of Medical Sciences, Wardha, IND; 2 Periodontics, Sharad Pawar Dental College, Datta Meghe Institute of Medical Sciences, Wardha, IND; 3 Conservative Dentistry and Endodontics, Guru Nanak Dev Dental College & Research Institute, Patiala, IND

**Keywords:** periodontics, titanium implant, fibronectins, repair, scaffold, cell behaviour, cellular fn, wound healing, ecm proteins, plasma fn

## Abstract

The formation of biomaterials is a physical phenomenon that is primarily influenced by the material's chemical and physical characteristics, as well as by the availability of proteins and their mutual interactions. A common extracellular matrix (ECM) glycoprotein called fibronectin (FN) is a biomaterial that is essential for tissue repair. Cellular FN (cFN), also known as the "large external transformation sensitive (LETS) protein" or "galactoprotein," was found during the quest for tumour markers twenty-five years ago and was later identified as the surface fibroblast antigen. Twenty different isoforms of the FN protein can be created by alternative splicing of a single pre-messenger ribonucleic acid (pre-mRNA) molecule. FN is an outstanding illustration of an ECM protein that intricately influences cell activity. FN is necessary for cell behaviours like cell adhesion, cell migration, and differentiation of cells as well as highly coordinated tissue processes like morphogenesis and wound repair. Plasma FN is absorbed by tissues and deposited in extracellular matrix fibrils along with locally generated cellular FN. cFN is produced by a wide range of cell types, including fibroblasts, endothelial cells, chondrocytes, synovial cells, and myocytes. FN and other cell adhesion proteins can promote cell attachment to tooth surfaces. Periodontal ligament (PDL) cell-ECM interactions, and consequently the regeneration of periodontal tissues, depends on FN. Specific FN segments serve as indicators of periodontal disease status and provide evidence for their potential involvement in the pathophysiology of the condition. FN is an all-purpose biomaterial that may be utilised for clinical applications ranging from tissue engineering to disease biology. Therefore, it would be desirable to develop materials that specifically bind to FN.

## Introduction and background

Protein adsorption from biological fluids, such as blood plasma, occurs shortly after a biomaterial has been implanted, mediating the interface between surface cells and the biomaterial. The composition of the adsorbed protein layer at the interface has a substantial impact on the nature of the tissue and material's fate, and it also has an impact on important aspects of cell response such as adhesion, spreading, migration, proliferation, and differentiation [1]. Protein availability and interactions between proteins that could lead to the adsorption of proteins that don't productively induce cells because of a deficient structure are two factors that drive the disorganized nature of the biomaterials process [2]. The selection of an appropriate biomaterial to provide a scaffold that replicates the native extracellular matrix and directs resident stem cells to restore functional tissue presents a significant challenge in tissue engineering (TE) [3]. A common extracellular matrix (ECM) glycoprotein called fibronectin (FN) is a biomaterial that is essential for tissue repair. The plasma form of FN circulates in the blood and, in response to tissue damage, it is absorbed into fibrin clots to influence the platelet function and mediates hemostasis [4]. FN promotes cell-ECM interactions during vital processes like development, wound healing, fibrosis, and tumour progression [5].

## Review

History of FN

In vivo, there are two distinct groups of FNs that have been identified as a result of quite diverse research projects. During post-World War II studies on the fractionation of human blood plasma, plasma FN (pFN), which is primarily found as a soluble dimer circulating in distinct body fluids, was identified as the first cold-insoluble globulin. Cellular FN (cFN), formerly known as the "large external transformation sensitive (LETS) protein" or "galactoprotein," was found during the quest for tumour markers 25 years ago. Later, it was identified as the surface fibroblast antigen [6]. By the middle of the 1970s, Vaheri and his colleagues united and chose a common name for the protein: fibronectin, which combines the Latin words *fibra*, which means fibre, and *nectere*, which means to bind or link. Reconsideration of the structural glycoproteins and ECM was driven by the discovery of FN. Most biologists had dismissed the ECM up to that point as a dull collection of inert, meaningless molecules [7].

Structure and biological activity of FN

**Figure 1:**
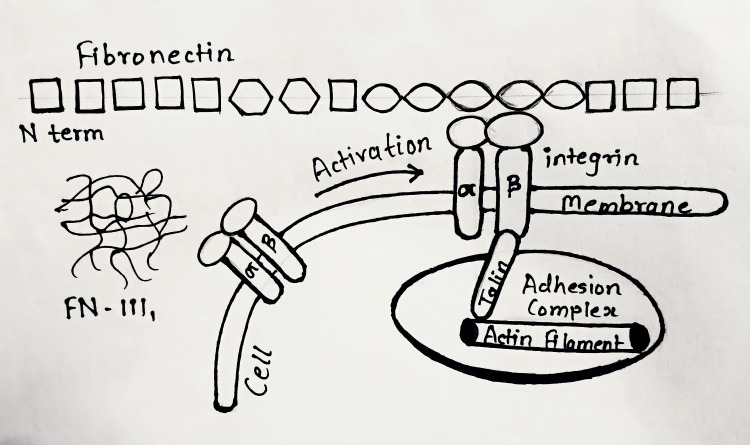
The cells bind and exert forces on fibronectin through transmembrane receptor proteins of the integrin family, which mechanically couple the actin cytoskeleton to the ECM via an elaborate adhesion complex structure of fibronectin bounded to integrin at the cell surface ECM: extracellular matrix; FN: fibronectin

In humans, FN has more than 20 distinct alternative splicing isoforms. Cold-insoluble globulin (CIG), or the physically complicated dimeric high-molecular-weight glycoprotein FN, is composed of two nearly identical subunits (250 kiloDalton) that are covalently connected by disulfide bonds adjacent to their C-terminal region [1]. The interactions between cells and the extracellular milieu that surrounds them determine how they behave. Cell receptors and integrins are primarily solicited because cells are highly sensitive to the mechanical and bio-physicochemical features of this ECM environment. Cells literally "feel" the distinctiveness of their surroundings through the integrins and react to important factors including mechanical characteristics. For instance, a lack of stiffness, such as that found in an extremely soft environment, impairs cell adhesion and, as a result, results in a lack of intracellular tension and an ineffective intrinsic signalling system [8]. By alternative splicing, 20 distinct isoforms of the FN protein can be produced from a single pre-mRNA molecule. FN domains contain binding sites for ECM substances like collagen, heparin, fibrin, and other FN molecules, as well as for cell binding via integrin receptors. FN is secreted in both its more soluble pFN and less soluble cFN forms [9]. Figure 1 shows how the structure of FN is bounded to integrin at the surface of the cell. FN fibrils exhibit significant elastic properties. Cells have the ability to stretch FN fibrils up to four times their relaxed length. The three repeats of FN-I, FN-II, and FN-III make up the majority of its multimodular structure, which gives the material its mechanical responses. Disulfide bonds are used to cross-link the beta-strands of type I and type II modules; type III modules do not include any disulfide bonds. The seven-beta strand sandwich motif, which is seen in many mammalian proteins, is present in the structural features of FN-III modules. It has been proposed that the unfolding of individual FN-III modules gives the FN fibrils their flexibility. Furthermore, by stretching FN-III modules, nucleation sites for the assembly of FN into fibrils that were previously concealed might be seen. The most plausible locations for these hidden binding sites, also known as cryptic sites, are the FN-III cores or the spaces between two close FN-III modules' hinge regions. FN-III modules contain extremely little sequence homology, with a typical sequence identity of under 20%, but having very comparable tertiary structures. The simulation results show that FN-III modules can be pre-stretched before encountering the main unfolding barrier by making only a few small adjustments to their tertiary structures [1,8,9]. 

FN as Cell Behaviour Conditioner

FN is an outstanding illustration of an ECM protein that intricately influences cell activity. It is a mediator in numerous cellular interactions with the ECM, possessing binding sites for integrins, other ECM proteins, growth factors, and itself. As a result, FN is essential for both highly coordinated tissue behaviours like morphogenesis and wound healing as well as cell behaviours like cell adhesion, migration, and differentiation. Growing data indicates that the selectivity of integrin engagement to this can lead to varied cell responses since FN can bind a variety of different integrins [10]. The first assembly of the basement membrane does not involve FN matrix assembly. This is because FN fibrils are one of the first ECM proteins to assemble during tissue development and wound healing. Additionally, FN molecules contain multiple domains that bind a variety of ECM proteins, growth factors, and small molecules [11]. Growth factors like platelet-derived growth factor (PDGF), Insulin-like growth factor (IGF-1), and transforming growth factor (TGF) are thought to be involved in periodontal regeneration and repair causing these cells to significantly upregulate the embryonic isoforms of fibronectin. The antigens that correspond to the fibronectin alternatively spliced variants do not reveal their functional activities, but the selective expression of these antigens suggests that they play very specific functions in periodontal regeneration and repair [12].

Properties of FN

The ECM contains proteins called FN that have a variety of cell activities, including structural support and signalling signals for gene expression, cell contractility, migration, and differentiation. Table 1 shows a list of various properties of fibronectin [1,4,5].

**Table 1:** Important characteristic properties of fibronectin

Properties of Fibronectin
Cell migration
Cell contractility
Differentiation of cells
Helps in growth factor signaling system
Signaling cue for cell growth
Possesses tissue-specific mechanical properties
serving as both a molecular reservoir and a structural scaffold

Types of FN

pFN

Disulphide (S-S) bond at the near C-terminus of pFN causes it to form a heterodimer that is made up of 230 kiloDalton and 235 kiloDalton chains [13]. Along with locally produced cellular FN, plasma FN is absorbed by the tissues and deposited in extracellular matrix fibrils. Given the plasma's abundance of FN, it has been thought that keeping plasma FN in a tight compact shape is essential to prevent abnormal interactions between FN protomers as well as between FN and other macromolecules and cell surface receptors [14].

cFN

cFN, a stretched and insoluble isoform that affects both ECM homeostasis and overall ECM-cell interactions, is secreted and organised into dense complex fibril networks. Increased cellular connections and the formation of fibril networks are made possible by the FN molecule's stretching [15]. Numerous cell types, including fibroblasts, endothelial cells, chondrocytes, synovial cells, and myocytes, develop cFN [16].

FN in wound healing conditions

The formation of ECM and re-epithelialization during wound healing depends upon FN. FN performs several functions in the healing of wounds as a result of the existence of numerous function domains and binding sites in its structure. It interacts with cytokines, the ECM, and many cell types. ECM formation is FN's primary function [17]. The early stages of wound healing are when pFN is most crucial since this is when it binds to platelets and fibrin, giving the fibrin clot more strength. Additionally, the FN in this clot is essential for the cell adhesion, migration, and aggregation of platelets. cFN is essential for the growth of granulation tissue and is added to the wound bed by fibroblasts and endothelial cells in the healing process [4]. By promoting platelet aggregation and adhesion to the damaged endothelium surface, FN reinforces the clot by creating a fibrin-FN network, also known as the "fibrin-fibronectin provisional matrix" [18].

Applications of FN in clinical dentistry

Because demineralization exposes collagen, to which FN binds, the role of FN in the attachment of cells to the tooth surface has received a lot of attention. FN and other cell adhesion proteins can make it easier for cells to attach to tooth surfaces [19]. FN can influence fibroblast attachment in the gingiva favourably in dental applications and implant operations, preventing inflammation-driven disintegration of the tissues around the implant [20]. It has been shown that covering titanium surfaces with FN and glow discharge plasma (GDP) increases the surface hydrophilicity, roughness, and ability to promote cell adhesion, migration, and proliferation [21]. To prevent dental epithelial cells formed from postnatal day-1 molars from adhering to an FN-coated dish, the cyclic arginine-glycine-aspartate (RGD) and a 1-integrin-neutralizing antibody were used for its prevention [22]. This suggests that FN-1 integrin interactions play a key role in dental epithelial-cell binding. FN production in dental pulp cells is stimulated by calcium ions produced from calcium hydroxide.

By coming into contact with other cells, FN may stimulate the development of dental pulp cells into mineralized tissue-producing cells, which are the primary cells responsible for forming dentinal bridges [23]. With a less significant impact on epithelial cells, FN coating of implant surfaces increased gingival fibroblast binding by two to three times [24]. The FN-immobilized chitosan scaffolds may work well as three-dimensional substrates for the attachment and growth of dental pulp stem cells. To stimulate odontogenic differentiation in the case of dental regeneration, they need to be supported by the proper biosignals [25]. Titanium has a remarkable affinity for human-FN (HFN) adsorption due to its hydrophilicity. Early cell adhesion, spreading, and proliferation are impacted by selective HFN adsorption [26]. The FN-conjugated, micro-grooved titanium (Ti) substrate is an effective surface to promote osteoblast formation and osteoblast marker gene expression in mesenchymal stem cells (MSCs) [27]. Root canal sealants showed biocompatibility and stimulated the expression of FN and tenascin (TN) [28].

Using a confocal laser scanning microscope and indirect immunofluorescence, it is possible to locate where FN was located in the dental pulp of developing and fully developed human teeth. Intense fluorescence was visible along the basement membrane in growing teeth that were exposed to the mesenchyme of Hertwig's epithelial sheath and freshly formed (mantle) predentine. The corkscrew-shaped FN fibres that crossed from the pulp into the predentine in a direction parallel to the odontoblasts' long axis could be detected between the cells as the odontoblasts becomes longer. In the odontoblast layer, fibronectin is present at every stage of dentogenesis. Von Korff fibres are fibrous structures between odontoblasts that are FN-positive. These structures are directly linked to dentinogenesis and odontoblast differentiation [1,12,13,18].

Applications of FN in periodontics

Periodontal ligament (PDL) cell-ECM interactions, and consequently the regeneration of periodontal tissues, depend on fibronectin [29]. Specific FN segments serve as indicators of periodontal disease status and provide evidence for their probable involvement in the pathophysiology of the condition [30]. Low quantities of FN fragments are present in healthy PDL sites. This is probably because of the tissue's high metabolic rate, which may result in a steady flow of ECM breakdown products in the form of fragments. Additionally, because FN is localised in the periodontal ECM, it can take part in tissue regeneration and wound healing. Type I and type III collagen fibrils in the PDL are coated with FN, and it also occasionally covers the gaps between neighbouring collagen fibrils and the cell membrane [29].

The fibrin glue system, a commercial product, includes FN, which promotes early wound attachment and healing [31]. A new approach to researching the impact of diabetes on periodontal disease is available through the efficient glycation of collagen type-I (COLI) and FN by methylglyoxal (MG) therapy. Protein glycation may have a role in the development of diabetes-related periodontal wound healing and the significant impact of glycated COLI and FN on human gingival fibroblasts (hGF) and human-periodontal ligament fibroblasts (hPDL) behaviour [32]. 

FN appears to have the potential to slow epithelial down growth, which in turn encourages root biomodification and regeneration. It is implicated in the attachment of gingival fibroblasts to root surfaces. The function of FN as a root biomodification agent in regeneration is discovered [33]. Citric acid and FN usage have the potential to encourage reattachment following periodontal therapy [34]. Faster healing results from surgery when FN and citric acid are used together. FN and citric acid together promote cellular proliferation [35]. The improved connective tissue attachment seen with tetracycline treatment alone appeared to be largely negated by the addition of fibronectin to tetracycline-treated roots [36]. When utilised as a regenerative therapy for intraosseous lesions in humans, enamel matrix derivative (EMD) and autologous fibrinogen or FN system (AFFS) combined with bovine-porous bone mineral (BPBM) had similar effects on decreasing periodontal probing pocket depth, clinical attachment loss, and defect fill [37]. The unique method of surface functionalization by FN deposition onto hydrolyzed polylactic-glycolic acid (PLGA) fibres is proposed to increase the bioactivity of scaffolds. According to the biocompatibility data, the FN deposition significantly enhanced both scaffold colonisation and cell behaviour. the impact of an FN gradient on electrospun surfaces, including the spreading behaviour and interactions between cells and scaffolds [38]. Connective tissue and alveolar bone regeneration increases after periodontal repair using guided tissue regeneration (GTR) surgery and results are somewhat improved by adjunct citric acid combined with autologous FN [39].

Diabetes-related chronic leg and foot wounds, as well as periodontal disease, frequently exhibit FN fragmentation. The theory that exposure to certain FN segments dramatically changes cell behaviour was further supported by cell culture assays [40]. Regardless of patient type, minimal FN could be found in the gingival crevicular fluid (GCF) of healthy sites. Additionally, the PDL's middle region is where collagen and new fibroblast production are both most active, indicating a connection between the locations of FN and fibroblast turnover. As a result, FN and its fragments are probably engaged in the interactions between cells and the ECM that support the preservation, regeneration, and wound healing of periodontal tissues [29,30]. An analysis of the sites in each group, however, showed that the concentration of FN in the GCF was highest in healthy sites and decreased when there was gingival inflammation. The concentration of GCF and FN was never to be physiologically or biologically active [41]. In the pathophysiology of Porphyromonas gingivalis fimbriae in adult periodontal disease, salivary FN is the key regulator [42].

Future directions

ECMs, both natural and synthetic, have been widely used in biomedical fields as one of the most successful elements in tissue regeneration [43]. The primary obstacle preventing the enrichment of biomaterials with FN or its fragments is the compound's high molecular weight, which limits its stability and bioavailability. Therefore, the development of substances that particularly bind FN would be desirable. Placing smaller molecules on scaffold surfaces would be a novel idea in this context and might be used to attract and retain FN from the extracellular environment [1]. FN is a versatile material platform used for a variety of purposes, including disease biology and tissue engineering, to finetune how an essential ECM protein is given to cells [44].

## Conclusions

The use of FN due to its cell behaviour conditioning has many applications in dentistry and periodontal therapy. Due to the structural support and signalling cues offered by FN, the ECM plays several roles in cell survival, migration, differentiation, gene expression, growth factor signalling, and cell contractility, among others. FN is working wonders in the field of dentistry, diagnosing periodontal disease. Specific FN fragments impair the activities of PDL cells in vitro, and FN fragments are identified in vivo in conjunction with periodontal disease. Specific FN segments serve as indicators of periodontal disease status and provide evidence for their potential involvement in the pathophysiology of the condition. The discipline of dentistry needs to use FN more in clinical aspects and more research on this adaptable biomaterial needs to be done. It controls cell behaviour and plays a key role in communication between the intracellular and extracellular environments, aiding in early attachment and healing of surgical wounds, and other functions that make it useful as well as a versatile biomaterial in dentistry and periodontal applications.
